# Unveiling Child Sexual Abuse Disclosure in China: An Ecological Exploration of Survivors’ Experiences

**DOI:** 10.3390/children11060688

**Published:** 2024-06-04

**Authors:** Tian Tian, Ilan Katz, Xiaoyuan Shang

**Affiliations:** 1 School of Counselling, Human Services, and Social Work, Faculty of Education and Social Work, University of Auckland, Auckland 1010, New Zealand; tian.tian@auckland.ac.nz; 2 Social Policy Research Centre, The University of New South Wales, Sydney, NSW 2052, Australia; ilan.katz@unsw.edu.au

**Keywords:** child sexual abuse, child disclosure, online, ecological analysis, China

## Abstract

Through a thematic analysis of firsthand posts from 258 abuse survivors in online forums from 2016 to 2023, this research examines the barriers that Chinese children encounter when disclosing sexual abuse. The anonymous narratives shed light on the motives behind survivors’ reluctance to reveal abuse, the outcomes following disclosure, and the wider implications for survivors and their families under culture. The findings underscore the need for early intervention upon disclosure, aiming to safeguard children from further harm and foster the development of an effective child protection framework.

## 1. Introduction

Child sexual abuse (CSA) has been recognised as a social problem globally, which can lead to severe immediate and long-term health and societal consequences [1]. Global meta-analysis studies indicated that the prevalence rate of CSA was estimated to be 12.7% in self-reported studies [2]. In China, the results of four published meta-analyses, encompassing 36, 27, 68, and 15 independent studies, respectively [3,4,5,6], indicate that the prevalence of CSA, including non-contact abuse, ranges from 9% to 18%. However, despite the high prevalence of CSA, most cases are not disclosed or reported in China. With a lack of official national statistics in China, we have to rely on media and NGO reports. According to the reports published by Protection of Girls (NGO), there were only 2952 cases of CSA publicly reported in the media from 2013 to 2021, with approximately 5500 child victims [7]. Additionally, Peking University’s “PKU Law” database recorded over 17,000 cases from 1991 to 2018 [8]. Both reports indicated a significant inconsistency between the relatively low official reports of CSA and the high prevalence reported in meta-analyses in China.

Research indicates a low rate of children voluntarily disclosing or reporting abuse, with a meta-analysis in the United States finding that over 95% of child sexual abuse cases are never disclosed to authorities [9]. Similarly, Tang’s (2002) [10] study found that 60% of child abuse victims and 68% of female victims did not disclose their abuse history to others in Hong Kong. In addition to children’s reluctance to disclose, parents in China also show hesitation in reporting suspected cases of CSA to authorities [11]. Additionally, Tan et al. [12] highlighted that 63% of child protection social workers refrain from reporting cases of child abuse to authorities. In order to increase child abuse reporting rates, China implemented a joint policy in May 2020, titled “Opinions on Establishing a Mandatory Reporting System for Cases Against Minors (for Trial Implementation)” [13], which stipulates that all professionals working with children, such as teachers, child welfare workers, and doctors, are mandated reporters. Additionally, the police department is responsible for responding to reports of CSA. However, the effectiveness of this policy in improving case reporting remains unevaluated.

Disclosure is critical as it marks the first step toward initiating interventions and obtaining support from family and formal services, which can greatly assist in the mental and physical recovery of victims [14]. However, the phenomenon of delayed disclosure or lifetime non-disclosure among child sexual abuse victims is an ongoing issue [15,16]. In recent years, numerous studies have pointed out that survivors are influenced by multiple factors when they choose not to disclose or delay disclosing the abuse. These factors include feelings of shame, guilt, self-blame, fear of not being believed, fear of negative social reactions, denial that the event was a crime, and a desire to avoid involving the authorities [17,18,19,20]. While the existing research is informative, past studies have relied on participants who voluntarily took part in research, potentially leading to issues of “unrepresented or underrepresented due to their unwillingness to participate” [21] (p. 479). Since the start of the #MeToo movement in 2018, there has been a surge of research internationally that explores the characteristics of child sexual abuse disclosure through the analysis of online posts [22,23,24], expanding the range of research participants in the study of this sensitive topic. However, in both online and traditional studies, most research is from Western countries and lacks diverse global results, especially limited research on disclosure in China.

The barriers to disclosing sexual abuse in China have been attributed primarily to cultural reasons. A vignette-focused group study in Hong Kong involving 12 children found that Chinese culture emphasises filial piety and loyalty to parents and elders, inhibiting children’s willingness to seek help when they experience abuse or neglect [25]. Furthermore, Xie [11] conducted interviews with 26 pairs of parents in Beijing and found that limited knowledge and awareness about sexual abuse, as well as cultural factors related to sexuality in Chinese society, inhibit parents’ receptivity to children’s disclosures and affect their willingness to report. This has also been supported by Sawrikar and Katz’s [26] research with minority ethnic groups in Australia (within the Chinese community), which indicated that mothers from a collectivist cultural background face greater pressure and risks, making them less likely to accept information child sexual abuse disclosures from their children. However, limited research explored the factors other than culture obstructing CSA disclosure in China.

In summary, this study aims to achieve the following:

- Explore the barriers to disclosing child sexual abuse in China;
- Reveal how specific socio-cultural factors influence child disclosure in China;
- Highlight the similarities and differences between this influence and international mainstream research.

This study aspires to provide insights and ideas for the prevention and intervention services for child sexual abuse within Asian communities and collectivist cultural influences.

### 1.1. Defining ‘Disclosure’

In discussing the disclosure of CSA by children and young people, it is important to define the concept of ‘disclosure’. This is not as straightforward as it might appear, with scholars adopting differing definitions in previous research. Consequently, research discrepancies have emerged due to the lack of a unified definition [27]. Disclosure was defined as reporting abuse to authorities by Gilligan and Akhtar [28], while Jones [29] offered broader definitions encompassing sharing experiences with others. Additionally, disclosure can be seen as a continuous process, often initiated by hesitant hints [30], indirect indications [31], or made by a third party [32]. Therefore, scholars generally categorise disclosure into occasional, deliberate, and induced forms [33,34]. However, Reitsema and Grietens [35] propose re-conceptualising disclosure as an interactive process between children and adults, where responses influence disclosure strategies. As pointed out by Allnock and Miller [36], despite attempts to disclose, young victims often faced unrecognised or disregarded disclosure attempts by adults. In summary, the development of disclosure definitions has progressed from focusing on the disclosure event to considering the process of disclosing and reflecting on disclosure outcomes.

For this article, we define disclosure as when a child or young person, who is a victim/survivor, informs a third party about the sexual abuse they experienced, or when the abuse is disclosed by another person. Disclosure can be sustained or transient, including occasional, deliberate, or induced disclosure. Additionally, the disclosure can be made by a third party, either directly by an observer of the abuse reporting it or indirectly by someone the victim/survivor confided in or who noticed changes in their behaviour.

### 1.2. Theoretical Framework

The ecological systems model was adopted to analyse the barriers to why victims did not disclose CSA, drawing from Bronfenbrenner’s [37] and Belsky’s [38] ecological theoretical framework initially used for understanding the interplay of factors contributing to child abuse. This framework can also be extended to the disclosure of CSA for a fuller understanding of child victims [37,39,40]. Based on in-depth interviews with 40 adult survivors, Alaggia [39] noted that personal, interpersonal, environmental, and contextual influences hinder and facilitate the ecological map of CSA disclosure. Furthermore, Collin-Vellian [15] identified three primary categories of barriers to disclosure, including internal barriers, barriers related to external factors, and barriers associated with the social environment, a classification closely aligned with the ecosystem framework. Recent systematic reviews of sexual abuse disclosure emphasise that the ecological systems model enhances our comprehension of individual, family, contextual, and cultural factors [41]. This model enriches our understanding of both the obstacles and facilitators in the disclosure process, although further data on cultural and contextual aspects are needed [41].

The ecological framework rests on the premise that individual behaviour can only be understood by taking into account factors at ontogenic, micro-system, exo-system, and macro-system levels [37,39,40].

Ontogenic development deals with the personal history and characteristics of the individual; micro-systems involve the immediate context of the individual (e.g., family and other intimate relationships); exo-systems refer to formal and informal structures such as neighbourhood, community, social networks and their availability; and macro-systems include cultural values and belief systems that impact on the individual [39,40].

By applying this framework, this article analysed the disclosure behaviours of victims of CSA from four levels: individual (ontogenic), family (micro-systems), extended families, neighbourhood and community (exo-systems), and cultural, value, and social factors (macro-systems), in order to understand the main obstacles that stopped victims of CSA disclosing abuse.

## 2. Methodology

### 2.1. Data Collection and Analysis

**Figure 1:**
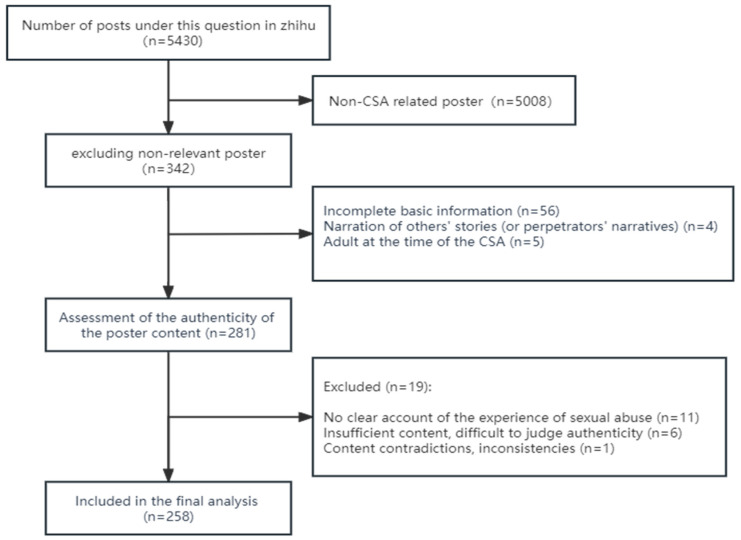
The flow diagram of the online posts selection.

This study is part of the Australia Research Council project “Protecting Sexually Abused Children in China”, approved by UNSW’s Ethics Committee. It utilises publicly available anonymous online data and does not require additional approval. The data analysed in this article are drawn from anonymous online community discussions (*zhi hu*, one of the most popular online chat communities in China) about the impact of CSA on victims’ sexual experiences in their adulthood. All posts were retained on the Zhihu platform under a particular question, and this question was open for indefinite replies unless removed by the question’s creator or the platform itself (https://www.zhihu.com/question/24000977, accessed on 1 March 2023). Therefore, the question’s creator has perpetual access to the responses, and other users can also view them. The research assistant conducted a review of 5430 responses posted between 26 December 2016 and 20 February 2023. The selection of the accounts was conducted jointly by two researchers. They screened posts based on the following inclusion criteria: (1) The discussion must relate to CSA. (2) Victims’ age of initial abuse must be below 18 years old. (3) The main part of any posts must be provided by the victims themselves and must be their own life experiences. (4) The posts included the main variables needed for analysis, which contained the time, place, form of victimisation, perpetrator characteristics, and the relationship between the victim and the perpetrator. In the initial screening, a total of 342 posts met the selection criteria. However, a key limitation of the analysis is the inability to track further and verify the accounts since the online accounts were anonymous or used pseudonyms. To ensure the data’s reliability, another independent researcher was invited to evaluate the reliability of the selected posts. The researcher assessed each account and categorised them as either “believe” or “not believe”, providing reasons for their “not believe” decisions. Then, all posts that were marked as “not believe” were collectively reviewed by all three researchers until a consensus was reached. Finally, 258 posts met the inclusion criteria and were included in the research analysis. The exclusion process can be viewed in the following (see Figure 1).

The study did not obtain individual informed consent; as Alaggia and Wang [22] noted, “Social media platforms that could be publicly accessed, without an account, were presumed to be open for public viewing” (p. 3). Thus, the use of public anonymous forum texts in this study is considered public data, and we did not request further ethics approval from the committee [21]. Additionally, this study utilised posts from the past nine years. Some original posters have left, and some posts were made in fully anonymous modes, so it is impossible to trace them back to specific individuals. Therefore, obtaining informed consent from the posters was not applicable in this study. We also made further efforts to protect user privacy by anonymising accounts; each post had the usernames removed and was assigned an anonymous label, such as “Poster 1”, to prevent any potential identification. The term “Poster” in this research refers to a forum user who posts online. A similar approach was adopted in several research works on online media forums, especially after the “MeToo” movement [21,22,24].

Thematic analysis was chosen to analyse the textual data from these 258 posts. This coding strategy was adapted from Braun and Clarke [42]. Initially, all posts were individually coded, with two researchers independently conducting open coding on the first 129 posts to identify themes. Following the first coding round, researchers clarified and discussed differences in codes and developed an initial coding framework to analyse the remaining content. As subsequent text analysis took place, new codes emerged. Through iterative coding development, refinement, and definition, researchers achieved data saturation, meaning no new information or themes emerged regarding the research questions presented.

### 2.2. The Characteristics of Victims and Perpetrators of CSA

Among the 258 victims identified, 220 were female and 38 were male, with the youngest victim being three years old. Approximately 77% of victims experienced sexual abuse before reaching the age of 12 (Table 1).

#### 2.2.1. Information of Victims

The majority of victims (76%, *n* = 197) were abused by a single perpetrator, while the remaining 61 victims faced repeated abuse by multiple perpetrators. Notably, a quarter of victims endured persistent abuse over an extended period. Female victims comprise 85% (*n* = 220) of the sample, with males representing 15% (*n* = 38).

#### 2.2.2. Information of Perpetrators

Table 2 provides details of the perpetrators as indicated in the online posts. The accounts referred to 353 perpetrators. It’s worth noting that there are more minor perpetrators (49.3%, *n* = 174) than adults (46.7%, *n* = 165).

The perpetrator profiles detailed in Table 2 reveal a gender disparity, with males accounting for the majority (93.5%, *n* = 330) of perpetrators. However, a small proportion (6.5%) of perpetrators were female, illustrating the importance of recognising that women can also perpetrate CSA. Acquaintances and relatives were the most common perpetrators, comprising 41.3% (*n* = 165) and 35.8% (*n* = 146) of all perpetrators, respectively. The posts describe various forms of sexual abuse, including indecent assault and acts meeting the legal definition of rape.

## 3. Findings

**Figure 2:**
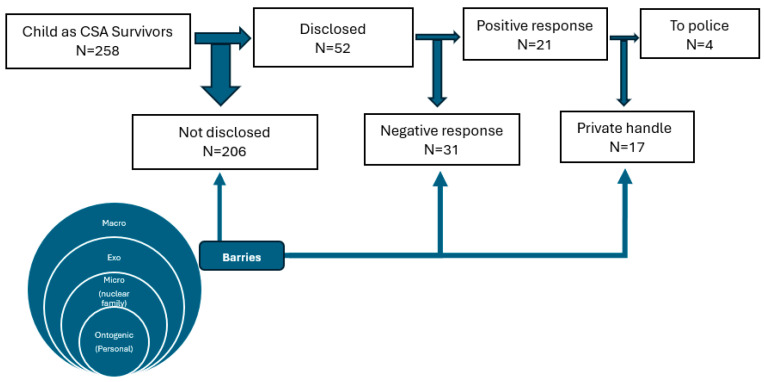
The process, barries and outcomes of CSA disclosure.

Figure 2 indicates the process of disclosure for these cases. This shows that of the 353 cases of sexual abuse reported in the online accounts, 294 (83%) cases were disclosed for the first time in this online forum, and just 52 cases had been disclosed previously. Of the 52 cases, less than half (*n* = 21) received a positive response and only 4 were reported to police. Consequently, this study explored the barriers hindering the disclosure of CSA in China. Furthermore, it also explored the negative responses to disclosed cases of child sexual abuse in China which may lead to further barriers to successful disclosure.

### 3.1. Barries to Disclose Child Sexual Abuse

This research identified four main themes with sub-themes categorised by the social-ecological framework, which contained the individual (ontogenic), family (micro-systems), extended families, neighbourhood, and community (exo-systems), and cultural, value, and social factors (macro-system). These themes are separate but not mutually exclusive.

#### 3.1.1. Barriers at Individual Level (Ontogenic)

The primary obstacles hindering children from disclosing sexual abuse, as frequently mentioned in the posts, predominantly revolve around individual-level factors. These multifaceted factors include (i) a lack of awareness/recognition of sexual abuse; (ii) children feeling unsafe and unprotected; (iii) children being incapable of disclosing sexual abuse.

##### A Lack of Awareness of Sexual Abuse

Many young victims lacked the maturity to understand the real meaning of sexual behaviours or their consequences. About a quarter of them were abused at a very young age and did not realise it until much later. Poster 64 was sexually assaulted at age 6 by her 10-year-old brother. However, she did not understand the meaning of these behaviours.

*“I did not tell my mum because I did not understand the meaning of his behaviours … I thought it was just normal (relationship with my brother). When I was 8 years old, I learned from school (physical hygiene education) that I should not have incest behaviours with my brother.”*

##### Children’s Feeling of being Unsafe and Unprotected

Children’s vulnerability puts them at high risk of sexual abuse as they can be easily manipulated and threatened by perpetrators. Children seldom choose to disclose abuse voluntarily, with some even trying to conceal the incidents for fear that others might find out. The online posts describe how children’s fear of disclosure manifests. For instance, Poster 121 highlighted a fear of further harm or revenge under threat. The victim was repeatedly bullied and molested at age eleven by her cousin at her aunt’s home; her cousin threatened her after the abuse, “*If you tell your family, you’re done!*”. Additionally, concerns about how disclosure might negatively affect the family were noted as a barrier.

*“… I was afraid that my parents would know … In a word, I was afraid that the people around me would be hurt, but I forgot that I was the injured one. At that time, I was afraid of causing trouble for my family.”*

(Poster 8)

Fear of not being believed or accused was another significant issue; Poster 159 underscores victims’ fear of not being believed by parents due to their traditional attitudes towards sexuality.

*“As I gradually became aware of sexuality, I dared not discuss it with my feudal, conservative, and stubborn parents. Whenever we watched scenes involving kissing in movies, they would always make disapproving remarks, making me too afraid to bring up the topic …”*

##### Children Incapable of Disclosing Sexual Abuse

Apart from a lack of understanding of sexual abuse, some children failed to disclose sexual abuse because they did not have sufficient language to describe the abuse they suffered. Poster 19 described how she was sexually abused by her cousin over a three-year period when she was aged between 6 and 8 years, while her cousin claimed he was giving her an “injection”. She felt uncomfortable and told her mother, “*My brother (cousin)2 often kisses me and hugs me*”. Her mother simply replied, “*That’s because your brother likes you*”. It was until some years later when she came across the word “rape” in a magazine that she understood what had happened to her. She described her emotional journey: “*However!!! As you become older, you gradually understand the implications of those behaviours and actions. This process of knowing the truth will feel like a bombshell! And overwhelmed with sorrow!!!… but you can’t talk about it now!!! It’s over. There was no third party present at the site*”.

#### 3.1.2. Barries at Micro-Systems: Families

The nuclear family is a significant factor influencing children’s behaviours and attitudes towards sexual abuse and disclosure. Data analysis revealed that CSA disclosure can be hindered by factors such as parents’ lack of awareness of sexual abuse, domestic violence, and parental separation or absence from children’s lives.

##### Parents’ Lack of Awareness

Many survivors’ posts highlight the lack of recognition by parents or guardians regarding physical and behavioural signals in sexually abused children, even including difficulties in interpreting ambiguous language cues.

*‘I actually feel repulsed by boys and have complete distrust towards them. However, my parents think that my lack of early romantic relationships is reassuring …’*

(Poster 142)

Furthermore, inadequate self-protection education from parents leaves children vulnerable to potential future abuse. For example, in Poster 80, the victim was sexually assaulted by her neighbour’s son in kindergarten and was once stopped by her mother, but “*[Her mother] failed to teach me the correct knowledge about sex, which led to me still not understanding it in the following years, while still being molested many times … [Parents’] sex education was a big failure!*”. 

##### Complex Family Environment

The complex family environment also had a significant impact on self-disclosure. Domestic violence is one factor that might stop children from disclosing CSA cases, which victims frequently mention. Poster 33, a woman raped as a child by her father, described how he also abused her mother: “*Why did I choose not to tell my mother? Because my mother often suffered from domestic violence from my father …*”. 

Tension between parents also has a negative impact on disclosure. Poster 126 mentioned how he stayed overnight with a schoolmate because his parents were “*always fighting each other; they never paid attention to me*”. As a result, he was raped a few times by a stronger schoolmate. He was threatened by the perpetrator, and he did not tell his parents. 

Problems caused by the divorce of their parents were also mentioned by some of the victims. For example, Poster 235 indicated that after the divorce of her parents, she felt “*a bit of low self-esteem and weakness, so they did not dare to resist … My parents divorced when I was five years old, and my mother brought me up by herself, and she was very busy with her work. … I don’t want to give my mum any trouble*”. 

Furthermore, some children were separated from their parents. In China in the middle of the 2010s, about sixty million children were living separately from their parents as left-behind children [43]. This was a common topic in the posts. The separation negatively affected children’s disclosure. We shall discuss this in the next section. 

#### 3.1.3. Barriers at Exo-Systems: Extended Families, Neighbourhood, and Community

Unlike micro-level factors, CSA perpetrators, whether relatives or acquaintances, often originate from this level. Consequently, obstacles at this level typically stem from interpersonal relationships and are closely intertwined with Chinese cultural norms.

##### Worries of the Extended Family Relationship

Analysing victim posts reveals the significant influence of extended family relationships on children’s disclosure behaviours. In China, the connection between nuclear and extended families holds great importance. During those CSA incidents that occurred within extended families, victims’ fear of deteriorating family relations between their nuclear family and relatives had a significant negative impact on children’s disclosing intentions. In Poster 36, the woman was raped by her cousin when she was 7 to 8 years old. She said that she “*pretended to be an ostrich for a long period of time, and didn’t want to do anything. It wasn’t until I became an adult that I dared to look directly at the past*”. She did not mention this experience to anyone because she was afraid of ruining the relationships. “*The two families still appeared to be intimate and friendly, and my uncles and aunts were very kind to me. I didn’t want to lose it, so I remained warm and polite to them*”. 

##### Worries of Neighbourhood and Acquaintance Relationship

Relational barriers to disclosure by children extend beyond the extended family level, even to the circle of familiar neighbours and family acquaintances. Some victims judged the proximity of the perpetrator’s relationship to the parent and the measure of the parent’s trust to decide whether or not to disclose. Poster 85 mentioned “*One reason for not telling mum and dad was that he was a good friend of my dad’s*”. Teachers were also seen as important acquaintances often believed by parents; according to Poster 51, the boy was touched in the private parts by a teacher: “*I didn’t dare to tell my family because my parents, like Fansiqi’s mother, would have blindly trusted the teacher and assumed that I was lazy and unwilling to study*”. 

##### Lack of Support from Neighbourhoods and Communities

The community settings also appeared to be important to disclosure, both in rural villages and urban neighbourhoods. The analysis found that the intention to disclose was negatively affected by a lack of empathy from neighbours and the community. Many of the victims were unable to receive timely help from neighbours. In Poster 18’s post, a woman described being bullied and sexually harassed by other children at school. She described her experience as

*“No one helped me. The teacher dared not intervene, my aunt would not take action for me, and my parents were far away. Feeling helpless, I chose to endure it without external help. If you couldn’t endure it, and decided to fight back, the bullying and sexual harassment would only get worse … Because I was a child that nobody cared about and nobody protected.”*

(Poster 18)

Poster 123 clearly demonstrates the vulnerability of left-behind children and the barriers for these children to disclose abuse:

*“All the villagers knew that I was a left-behind child, many of them teased me: Your parents abandoned you. I was very young at that time, so I believed what they said. After I was raped, the perpetrators always threatened me: If you tell anyone, your parents will never come back, they also made me confirm that I would never tell anyone.”*

#### 3.1.4. Barriers in the Macro-System: Cultural, Value, and Social Factors

At the macro-system level, barriers include the stigmatisation of victims in the broader culture and media, patriarchal attitudes, the sense of shame or ‘face’, and fear of damage to reputation, which are characteristic of Confucian cultures such as China.

‘Son preference’ has become one of the obstacles. Poster 135 was a girl aged 6 to 8 when she was abused. She was fostered in her paternal extended family home with grandparents and a cousin every summer and was sexually abused by her cousin during those visits. The victim described how she could not disclose the abuse: “*I couldn’t even cry—if I cried, the cousin would not be blamed, but I would be beaten because grandparents looked up to boys and down on girls. So I always kept silent*.”

Furthermore, societal conservatism regarding sex and the stigmatisation of victims of sexual abuse causes victims to fear damage to their personal reputation, leading to ridicule or exclusion by peers.

*“I worry that he (the perpetrator) might expose this, tarnishing my reputation… After it’s exposed, I’ll be the only one suffering. Society is really unfriendly towards women.”*

(Poster 260)

Because of the stereotype of chastity in Chinese culture, some female victims described internalising shame and self-criticism: “*I felt ashamed of myself. I don’t know why I felt that way. I didn’t tell anyone about it, including my family members.*” (Poster 38).

This was evident in a very small proportion of cases, mainly in cases of female-on-female sexual abuse and male victims of female sexual abuse. This phenomenon is another manifestation of cultural stereotypes, where males are considered the initiators of sex, with females engaged as passive participants. Therefore, where a perpetrator of CSA is female, and the victim is male, some male victims believe that they are not harmed. For example, “*I never talked to adults about this … After all, it didn’t cause much harm*” (Poster 279). Alternatively, some male victims regarded it as a sexual game and concluded that it was “fun” (Poster 119 and Poster 151).

### 3.2. Response to Disclosed and Exposed Cases of Child Sexual Abuse

Families responded differently after child disclosure. Voluntary disclosure (self-disclosure) by children plays a vital role in initiating legal processes and conducting therapeutic interventions in cases of CSA [44]. In 38 cases (10.7% of 353), children voluntarily disclosed (or delayed disclosure) their abuse experiences to family members or friends. As indicated by Table 3, in most cases, children made disclosures to direct caregivers, especially female caregivers, including mothers, grandmothers, and sisters; furthermore, fathers would also be the primary disclosure choice.

#### 3.2.1. Response to Disclosure

Regardless of the form of disclosure, in most cases of disclosure (*n* = 26), children faced actual negative feedback, hindering their ongoing disclosure or formal reporting (Table 4). This feedback included laughing at the victim, scolding them, taking the perpetrator’s side, not believing them, and providing no response.

*“I told my dad on the spot, but my stepmother scolded me, and my dad said you must have misunderstood.”*

(Poster 62)

In some cases, families even requested children to keep the abuse secret, as mentioned by Poster 259: “*Mom told me not to tell dad, afraid he would be upset*”.

Another response (*n* = 5) involved comforting the victim but ultimately choosing not to pursue further action, often due to the perpetrator being a family friend or relative. For instance, Poster 155 mentioned, “*(Mother) told me to keep quiet, not to make a fuss, and comforted me that everything was over*”. Poster 201 stated, “*My mom comforted me superficially, but she seemed uncomfortable psychologically; however, (the perpetrator) is her brother-in-law, so it’s hard to speak up*”. Thus, in both types of responses, the reactions to disclosure were either ineffective, re-traumatising, or both.

Families in the posts tended to handle disclosures of sexual abuse through informal channels rather than reporting to law enforcement. Ten posts mentioned family members choosing to confront or scold the perpetrator directly. For example, Poster 80 mentioned, “*My dad was furious when he found out, and asked me to contact that scumbag to teach him a lesson*”.

However, not all confrontations with the perpetrator provided effective support for the victim. For instance, according to Poster 237, after the child had been sexually assaulted by a neighbour and informed the parents, “*They (parents) were very angry and called several people, my uncle holding a rope*” “*Angry said they want to kill him*” *… The scene was a bit chaotic,* “*T (the perpetrator) said he would marry me in the future… Later, they heard that my hymen was not broken, it was like a relief, many people came to persuade, and then the matter was dropped*”.

In seven cases, after disclosure, although the family did not punish the perpetrator, they prevented the perpetrator from approaching or making contact with the child again. For example, according to Poster 76, after being sexually abused by their mom’s boyfriend, “*the next day I told my mom, she told me to lock the door at night, and I never saw that man again*”.

#### 3.2.2. Disclosure and Judicial Intervention

The analysis of the online accounts suggests that few cases of CSA received an effective response or intervention after disclosure. Only four cases (1.1%) were reported to the police, three of which resulted in the prosecution of the perpetrator. However, in one case, the perpetrator could not be prosecuted due to a lack of physical evidence.

*“my grandfather was kept at the police station for half a day, but because I had no visible injuries, it didn’t even qualify as a criminal case, allowing him to escape justice.”*

(Poster 86)

Reporting CSA to the authorities involves a ‘reporting cost’ [25] as indicated by some victims in the posts who expressed a desire to report but ultimately refrained due to lack of evidence, family relationships, or societal pressures.

*“If I had seen answers on Zhihu about collecting evidence in rape cases or something similar earlier, maybe I would have reported”*

(Poster 135);

*“I remember my own aunt kneeling in front of my mother begging her not to report”*

(Poster 240);

*“I wanted to, but I was too scared, too scared to report… the public opinion on this matter is too harsh, as a victim, I don’t have the strength to endure any more harm”*

(Poster 33).

#### 3.2.3. Response Depend on Kinship Relations

Another emerging theme is that in cases of kinship abuse, the relationship between the perpetrator and the disclosed parent/guardian influences the family’s response. Among the 22 cases of familial abuse disclosed, the parent to whom the children disclosed often sided with their immediate relatives or partners rather than with the children. For example, Poster 25 informed her mother about the sexual assault by her uncle (her mother’s brother). However, her mother did not believe her and sided with the uncle. The father, on the other hand, believed the victim and beat the uncle. Furthermore, if both parents were aware, it could lead to division among extended family members, creating opposing factions within the families. It is noteworthy that these actual negative reactions align with the survivors’ anticipated concerns and barriers mentioned earlier.

## 4. Discussion

This research delves into the complex and difficult process for child victims to disclose their child sexual abuse experiences in China (Figure 2). According to social ecological systems theory, individual behaviour can only be understood by taking into account how the individual, family (micro-system), community (exo-system), and cultural and institutional (macro-system) levels affect the willingness of children to disclose CSA and the likely response to those disclosures. The findings reveal obstacles to CSA disclosure existing across these levels, with interconnected factors. Moreover, negative responses following a child’s disclosure hindered the child’s ability to successfully disclose, reinforcing their concerns and acting as another major barrier. Furthermore, the family’s choice to handle these cases privately has hindered formal reporting opportunities.

This research at the individual level highlights that a considerable number of victims (approximately a quarter of victims) were initially unaware that they had experienced sexual abuse. This finding supports earlier research, indicating that Chinese children lack basic knowledge and self-protection skills to prevent abuse [45]. As several systematic reviews have pointed out [46,47], mainland China’s formal social services and support systems for preventing CSA are still inadequate. Raising awareness about CSA and engaging in conversations about it continue to be highly challenging due to sexual shame and societal pressures in China [46]. Over the past three decades, sex education in China has primarily targeted adolescents in developed areas, and to date, sex education is not mandatory in China’s nine-year compulsory education system. Current sex education prevention programs are driven by non-government organisations, as highlighted by Wu and Zeng [47]. It is evident that the lack of sex education, especially for younger children who are more vulnerable to sexual abuse, is one of the significant reasons why many children fail to recognise sexual abuse or know how to respond to inappropriate sexual behaviour. Although educational policies are macro-level systemic factors, they also influence micro-level systems.

The lack of sex education is evident not only among children themselves but also among parents in this study. Several cases in this study illustrate how parents’ lack of awareness of sexual abuse makes it difficult for them to identify it based on their children’s vague descriptions or abnormal behaviours, resulting in child sexual abuse going undetected or unreported. This finding aligns with previous research, indicating that Chinese parents lack relevant knowledge about CSA prevention, particularly concerning the characteristics of offenders, sexual abuse of boys, and non-physical consequences of CSA [11,48,49]. Moreover, most CSA prevention programs in China currently focus on the individual level through school education, with limited involvement of parents in CSA prevention programs [46]. Therefore, it is imperative to consider incorporating parents of children into the target group for CSA knowledge training in China [11,49].

Another important finding at the family level is that families often fail to provide appropriate support for children who have experienced sexual abuse. In China, the nuclear family is gradually being separated from the extended family [50]. However, analysis of these accounts reveals that neither nuclear nor extended family members were consistently able or willing to protect victims/survivors of CSA upon disclosure. Out of 353 cases, only 25 received an effective response according to the victim/survivor’s perspective. This analysis of anonymous online accounts suggests that there were three main factors influencing parents’ intention to support children and report the cases: the degree to which the children were believed, the degree of injury caused by perpetrators from parents’ understanding, and how disclosure would affect the relationship between parents and perpetrators.

This research confirms that children may not only fear not being believed when they disclose abuse, and that many also actually experience disbelief from their parents. Previous studies on parents of preschoolers in Beijing have highlighted this issue, revealing that over half of parents doubt the credibility of their children’s reports of abuse [25,51] and finding that Chinese parents struggle to determine which accusations from children should be believed. This parental distrust creates a cycle where children may face re-victimisation during the disclosure process, a phenomenon also observed in studies conducted in Zimbabwe and South Africa [52,53]. Moreover, child victims in this study express profound frustration and injustice due to the lack of belief and support from their families, potentially leading to significant psychological harm.

There is a lack of consensus among Chinese parents regarding the definition of CSA. In our posts, some families believed that only rape (penetrative sex) was considered serious enough, while other forms of sexual abuse behaviours were not deemed criminal offences. Consequently, they were reluctant to report the abuse or involve the police, fearing that it would not result in protection for the victim and would bring shame to the family. However, in Chinese criminal law, the definition of rape of a minor (under 14 years old) is based on the contact approach (where there is any contact of sexual organs between the perpetrator and a female victim), while rape of women is deemed through penetrative sex [54]. It seems the confusion regarding the definition of child sexual abuse might stem from a lack of understanding of relevant legal provisions for children and the lack of public legal education.

The ‘guanxi’ (relationship) dynamics between perpetrators and families significantly influence the disclosure and reporting choices of both victims and families in China, impacting not only the familial but also the exo-system levels. Despite the majority of sexual abuse cases being perpetrated by acquaintances or relatives, with 88% of perpetrators falling into this category in our study, Chinese families often perceive child sexual abuse (CSA) as being primarily perpetrated by strangers [55]. This perception is rooted in the belief that “family members” are unlikely to harm children [11,49], encompassing not only close relatives but also familiar acquaintances within the community. Some children also avoid disclosing abuse due to the fear of disrupting adult interpersonal relationships, as the ‘guanxi’ is essential to form the ‘Chaxu geju’ (Differential Mode of Association) (‘Chaxu geju’ was a concept to explain the Chinese social relations structure established by Xiaotong Fei, which means that the social structure in China differs from the Western model. In China, it is not a neat bundle of sticks but rather like ripples spreading out from a stone thrown into water. Each person is the centre of the circles of influence created by their social connections. The so-called public sphere in China actually originates from the expansion and transformation of the private sphere or is dominated by it.) in China [56] (p. 34). This trust extension might result in risks posed by relatives, friends, and teachers being overlooked and underestimated.

Furthermore, in cases where the perpetrators were acquaintances or relatives, family members often chose to handle them privately, such as using violence or intimidation, rather than through formal channels. Only four cases were reported to police in our research. Such reluctance to involve external authorities stems from a lack of trust in the justice and law enforcement systems [11], as well as a cultural resistance to external interference in familial affairs [57]. The reluctance to seek state intervention is evident not only in family decisions but also in the reporting tendencies of professionals [12].

Peers were the second most common category of perpetrators of CSA, but the risk they posed was generally overlooked, which also hindered the disclosure of sexual abuse cases. As noted above, juvenile perpetrators were as numerous as adult perpetrators in this research. International prevalence studies also suggest that sexual abuse against minors may be committed more frequently by peers than by adults [58]. This may be because minors spend more time with their peers and adults often ignore the risk of sexual abuse posed by peers.

At the macro-system level, the current research reaffirms previous findings indicating that cultural values can influence the disclosure of child sexual abuse (CSA) [59]. These cultural barriers encompass ignorance, fear, and stigma surrounding sexuality, patriarchal traditions, and the prioritisation of family reputation over children’s safety in Chinese culture [11,49,57]. This study suggests that cultural factors subconsciously impact barriers to disclosure faced by children and families on other levels. However, it is crucial to note that cultural influences are not unidirectional; cultural factors may also facilitate disclosure. For instance, supervision within extended families and informal networks may serve as protective factors for children, as found in previous research [11,60].

Furthermore, past research suggests that male children who are victimised by females may suffer more severe harm because they may feel manipulated or that their masculine self-image has been compromised [57]. However, this study found that males are more likely to perceive such experiences as games and do not see themselves as victims. The traditional cultural emphasis on female chastity and perceived benefits of male sexual behaviour may lead males to perceive themselves as beneficiaries of sexual events, potentially undermining the victim’s identity. This may need further exploration.

This research highlights institutional barriers as another significant aspect of the macro-system in China. Firstly, the absence of an accessible child protection system is evident in the scenarios depicted in the posts. In most cases, the only authority available for children or families to disclose to was the police, who were not expected to respond unless the abuse was severe. Many posts described a sense of hopelessness about receiving support if they reported the abuse. Although Chinese laws clearly indicate the need to protect minors, including from sexual abuse, there is a lack of supporting organisational structure and effective resource allocation for implementing these laws. Key stakeholders, such as teachers, doctors, and social workers in mandatory reporting systems, were not mentioned in the posts. This absence underscores the need for a comprehensive child sexual abuse protection system, as previously noted in Shang’s research [54] and further emphasised in this study. Developing and improving the child protection intervention system will be a critical area for future research in China.

### 4.1. Conclusion and Policy Implications

This study employs a new approach to analysing posts on Chinese social media, expanding research avenues on the relatively inaccessible topic of CSA in China. The 258 posts provide insight into the tip of the iceberg of CSA issues, highlighting the challenges children face in disclosing and reporting abuse. Key obstacles include the lack of awareness about sexual abuse, the prioritisation of adult relationships over child protection in cultural values, and the flawed and distrusted protection system.

Therefore, this paper suggests, firstly, the urgent need for universal CSA prevention education integrated into primary and secondary school curricula, not solely provided by NGOs. Parents should also receive education on recognising, reacting to, and reporting abuse, especially within extended family and peer contexts. Secondly, altering traditional value systems to enhance child protection is a long-term process. Given the sensitivity of CSA in Chinese society and the disclosure process’s obstacles, the internet can be a valuable tool for survivors to disclose their experiences. Social networks provide a platform for survivors to share information and gain empowerment when traditional systems do not support them [4,61]. Lastly, China must implement current child protection policies, such as the 2021 Mandatory Reporting Policy, effectively. This entails training more professionals to ensure timely and confidential reporting, gradually building trust in the government system among children and families.

### 4.2. Limitations and Significance of the Research

The analysis of CSA victims’ accounts in anonymous online communities sheds light on their disclosure experiences and responses. However, these findings have limitations. Firstly, they do not represent all CSA survivors, as the sample was drawn from a single online community, and not all victims use such platforms or are willing to share their experiences. The relationship between the sub-population who discussed disclosure online and the population of victims/survivors is unknown. Secondly, the accounts are subjective and unstructured expressions of individuals’ memories, lacking comprehensive life course information. Thus, the analysis relies on partial and subjective data, limiting further interrogation by researchers. Moreover, these accounts may be influenced by other life experiences, which cannot be factored into the analysis. In China, the majority of CSA cases have neither been disclosed nor reported to authorities. This makes it difficult to obtain samples of CSA cases except via the judicial system. Despite its limitations, this research offers an innovative approach to understanding CSA disclosure experiences in China. This study reveals the multiple barriers faced by CSA victims in disclosure, filling a gap in the international literature on China. It emphasises the importance of understanding the unique social, cultural, institutional, and familial factors influencing disclosure behaviours. These findings provide a foundation for more effective prevention and intervention strategies in similar contexts. While this study contributes valuable insights, caution is warranted in generalising the findings to the broader population of CSA survivors.

## Figures and Tables

**Table 1:** Profiles of the victims: age, sex, and age of initial abuse.

Age of Initial Abuse		Gender and Number		Number of Perpetrators and Victims		Instance of Abuse	
Time	N		N	Perpetrators (N)	Victims (N)		N
3–6 years	66	Male	38	One	197	Once	95
7–12 years	133	Female	220	Multiple	61	Repeated	163
13–15 years	29						
16–18 years	4						
Unknown	26						
Total	258		258		258		258

**Table 2:** Profiles of perpetrators.

Age Group		Gender		Relationship to Victims	
	N		N		N
Adults	165	Male	330	Strangers	42
Minors	174	Female	23	Acquaintances	165
Unknown	14			Relatives	146

**Table 3:** Cases in which CSA was voluntarily disclosed by children.

Identity of Perpetrator		To Whom Abuse Was Disclosed	
	N		N *
Relatives	22	Mother	25
Acquaintance	13	Father	12
Stranger	3	Grandmother	5
		Sister/brother	2
		Friend/boyfriend	3
		Teacher	2
		Stranger	1

* N denotes that when abuse was disclosed to multiple individuals, such as parents, there was crossover present.

**Table 4:** Different responses after disclosure.

Response after Disclosure	Voluntary Disclosure	Passive Disclosure		In Total
	Self-Disclosed Cases	Caught in The Act	Other Informed	
Calling the police	2	2	0	4
Directly beating or scolding the perpetrator	7	3	0	10
Prohibiting the perpetrator from contacting the victim	4	2	1	7
Comforting the child, but took no further action (seen as negative as well)	5	0	0	5
Providing no support (contains laughing at the victim, standing on the perpetrator’s side, not believing, scolding the victim and no response)	20	3	3	26
Total	38	10	4	52

## Data Availability

The study data are anonymously available online on an open platform (https://www.zhihu.com/question/24000977, accessed on 1 March 2023). They were retained under a specific question, which remained open for indefinite replies unless removed by the question’s creator or the platform itself.
